# Relationship Between PTEN and Angiogenesis of Esophageal Squamous Cell Carcinoma and the Underlying Mechanism

**DOI:** 10.3389/fonc.2021.739297

**Published:** 2021-11-02

**Authors:** Chenbo Yang, Chao Chen, Qiankun Xiao, Xiaoqian Wang, Yuwei Shou, Xiangyu Tian, Shuaiyuan Wang, Hui Li, Yinghao Liang, Jiao Shu, Kuisheng Chen, Miaomiao Sun

**Affiliations:** ^1^ Department of Pathology, The First Affiliated Hospital of Zhengzhou University, Zhengzhou, China; ^2^ Henan Key Laboratory of Tumor Pathology, Zhengzhou University, Zhengzhou, China

**Keywords:** esophageal squamous cell carcinoma, tumor-associated macrophages, PTEN, PI3K/AKT signaling pathway, angiogenesis

## Abstract

Esophageal squamous cell carcinoma (ESCC) has high morbidity and mortality rates owing to its ability to infiltrate and metastasize. Microvessels formed in early-stage ESCC promote metastasis. Phosphatase and tensin homolog (PTEN) mediates macrophage polarization, but its effect and mechanism on early ESCC angiogenesis are unclear. To explore the molecular mechanism underlying early ESCC metastasis through blood vessels, we investigated the relationship between PTEN/phosphoinositide 3-kinase (PI3K)/p-AKT protein levels, number of infiltrated macrophages, and angiogenesis in ESCC and ESCC-adjacent normal esophageal mucosa tissues from 49 patients. Additionally, PTEN was overexpressed or silenced in the esophageal cancer cell line EC9706, and its supernatant served as conditioning medium for M1 tumor-associated macrophages (TAMs). The culture medium of macrophages served as conditioning medium for esophageal tumor-associated vascular endothelial cells (TECs) to study the biological behavior of PTEN-plasmid, PTEN-siRNA, and control TECs. We found that M1 TAM infiltration in ESCC tissues was low, whereas M2 TAM infiltration was high. Microvessel density was large, PTEN was down-regulated, and the PI3K/AKT pathway was activated in ESCC specimens. These parameters significantly related to the depth of tumor invasion, lymph node metastasis, and pathological staging of ESCC. Silencing of PTEN in EC9706 cells significantly activated the PI3K/AKT signaling pathway in macrophages, promoting M1-to-M2 TAM polarization and enhancing TECs’ ability to proliferate, migrate, invade, form tubes, and secrete vascular endothelial growth factor. We believe that PTEN silencing in esophageal cancer cells activates the PI3K/AKT signaling pathway in macrophages *via* the tumor microenvironment, induces M2 TAM polarization, and enhances the malignant behavior of TECs, thereby promoting ESCC angiogenesis. Our findings lay an empirical foundation for the development of novel diagnostic and therapeutic strategies for ESCC.

## Introduction

The 2020 Global Cancer Report indicated that esophageal cancer ranks seventh among all tumors in terms of incidence and sixth in terms of mortality (1). The incidence of esophageal cancer shows significant regional differences. For example, the incidence of esophageal cancer in China is among the highest in the world, and the five-year overall survival rate is low (approximately 35%) (2). Pathological blood vessel growth is a typical sign of solid tumors and a necessary condition for tumor growth and metastasis. Because new blood vessels provide essential nutrients for tumor cell growth and metastasis, and tumor cells can induce the formation of new blood vessels. Esophageal squamous cell carcinoma (ESCC) has strong ability to infiltrate and metastasize. Microvessels are formed in the early stage of the tumor, which promote the metastasis of cancer cells. Although the tumor infiltrates only the superficial esophageal tissue, 50% patients show local metastasis under the esophageal mucosa (3). However, the pathological mechanism of early metastasis of esophageal cancer remains unclear.

Macrophages are infiltrating immune effector cells that account for the largest proportion of immune cells in the tumor microenvironment. They are called tumor-associated macrophages (TAMs) in tumor tissues and play an important role in the occurrence and development of tumors (4). TAMs are mainly divided into M1 type macrophages (M1 TAM), which activate the body’s tumor immune response, and M2 type macrophages (M2 TAM), which promote tumor growth (5).

Phosphatase and tensin homolog (PTEN) is a well-known tumor suppressor and an important negative regulator of the PI3K/AKT signaling pathway. Loss of function of *PTEN* promotes tumor malignancy (6). Some scholars have found that PTEN down-regulation in pancreatic and lung cancer can regulate the polarization of TAMs to the M2 type by activating the PI3K/AKT signaling pathway, thereby promoting tumor angiogenesis and inducing tumor migration and invasion (7, 8).

Previous studies have demonstrated infiltration of large numbers of M2 TAM in ESCC, which is related to the depth of invasion, lymph node metastasis, and tumor-node-metastasis (TNM) staging, and can promote formation of tumor blood vessels and lymphatics by secreting vascular endothelial growth factor (VEGF)-A and C (9). However, the polarization factors and the mechanisms *via* which TAMs drive ESCC malignancy remain unclear. In this study, we aimed to investigate the mechanism of PTEN-induced TAM polarization and its effects on tumor angiogenesis, providing a theoretical basis for further studies on the mechanism of ESCC angiogenesis.

## Materials and Methods

### Tissue Samples

From 2018 to 2020, esophageal cancer tissue samples and normal esophageal mucosa tissue samples adjacent to cancer were collected from 49 patients from the Department of Pathology of the First Affiliated Hospital of Zhengzhou University. All cancer tissues were found to be ESCC after the pathological diagnosis. None of the patients underwent radiotherapy, chemotherapy, or immunotherapy before surgery, and the cancer tissues and adjacent tissues were routinely dehydrated and embedded in paraffin after surgery. The 49 patients included 32 men and 17 women, aged 47−83 years. According to the 2017 TNM staging standard for esophageal cancer (eighth edition) jointly issued by the Union for International Cancer Control and the American Joint Committee on Cancer, among the 49 ESCC cases, 32 were in stage I–II and 17 were in stage III–IV. All patients participating in the study provided informed consent, which was reviewed and approved by the Ethics Committee of the First Affiliated Hospital of Zhengzhou University.

### Cell Lines and Cell Culture

Human ESCC cells (EC9706) were a gift from the State Key Laboratory of Molecular Oncology, Institute of Cancer Research, Chinese Academy of Medical Sciences. The human monocytic leukemia cells (THP-1) were purchased from Shanghai iCell Bioscience Co., Ltd. (Shanghai, China), and human umbilical vein endothelial cells (VECs) were purchased from Suzhou Beina Chuanglian Biotechnology Co., Ltd. (Suzhou, China). Fetal bovine serum (10%; Gibco, Grand Island, NY, USA) and a 1% penicillin-streptomycin mixture (Solarbio Science & Technology Co., Ltd., Beijing, China) were added to basic Dulbecco’s modified Eagle’s medium (DMEM; Gibco) and Roswell Park Memorial Institute (RPMI)-1640 medium (Gibco) to obtain the complete medium. EC9706 and VECs were cultured in complete DMEM and THP-1 cells in complete RPMI-1640 medium.

### Immunohistochemistry

The streptavidin-peroxidase linkage method (SP method) was used for immunohistochemical staining per the instructions of the universal SP kit (Zhongshan Golden Bridge Biotechnology Co., Ltd., Beijing, China). After sections were deparaffinized, the high-pressure thermal repair method was used for antigen retrieval. Endogenous peroxidase was inactivated with 3% H_2_O_2_ for 20 min, and then sections were blocked with 10% normal goat serum for 20 min and incubated with primary antibodies overnight at 4°C. Next, sections were incubated with biotin-labeled goat anti-rabbit/mouse IgG for 10 min, followed by horseradish peroxidase-labeled streptavidin incubation for 10 min. Between each incubation, sections were washed with phosphate-buffered saline (PBS) three times, 3 min each. The following antibodies were used to detect the antigen: anti-CD16 rabbit polyclonal antibody (1:200; 16559-1; Proteintech Group, Rosemont, IL, USA), anti-CD163 mouse monoclonal antibody (1:200; ZM-0428; Zhongshan Golden Bridge Biotechnology Co.), anti-CD31 mouse monoclonal antibody (1:200; ZM-0044; Zhongshan Golden Bridge Biotechnology Co.), anti-PTEN rabbit monoclonal antibody (1:2000; ab267787; Abcam, Cambridge, UK), anti-PI3K mouse monoclonal antibody (1:300; 67121-1; Proteintech Group), and anti-phospho-AKT (p-AKT) rabbit monoclonal antibody (1:200; 4691; Cell Signaling Technology, Boston, MA, USA). The DAB chromogenic solution was added dropwise to the sections and incubated for 3 min, followed by counterstaining with hematoxylin and mounting on the slide after dehydration. The positive control group included a tissue section with known positive expression of CD16, CD163, CD31, PTEN, PI3K, and p-AKT; the negative control group was treated with PBS instead of the primary antibody.

CD163 is a type I membrane protein that can be used as a surface marker of M2 TAMs, CD16 is a marker of M1 TAMs, and CD31 is a membrane glycoprotein frequently used for the diagnosis of tumor VECs and for determining microvessel density (MVD). CD16, CD163, and CD31 positivity is indicated by uniformly stained brown particles on the cell membrane, PTEN positivity by uniformly stained brown particles in the nucleus or cytoplasm, and PI3K and p-AKT positivity by uniformly stained brown particles in the cytoplasm. Counting was performed as described previously (10–12). The entire slide was observed under a low-power lens of a microscope (BX5; Olympus, Tokyo, Japan) and five representative hot spots were selected. A high-power lens (400×) was used to count CD16, CD163, and CD31 positivity in the five hot spots. The average counts of the five hot spots in each slice are the densities of CD16, CD163, and CD31. The median of each index was defined as the cutoff value, which was divided into high-density and low-density groups. A low-power lens (200×) was used to score the staining of PTEN, p-AKT, and PI3K in the five hot spots. The final score was obtained by multiplying the staining intensity with the proportion of positive cells. The staining intensity was divided into missing, weak, medium, and strong, which were recorded as 0, 1, 2, and 3 points, respectively; the proportion of positive cells was divided into 0–5%, 6–25%, 26%–50%, 51–75%, and 76%–100% categories, which were recorded as 0, 1, 2, 3, and 4 points, respectively. The average score of the five hot spots in each slice indicated the expression of PTEN, p-AKT, and PI3K; those with 0–4 points were placed in the low expression group, while those with 5–12 were placed in the high expression group.

### Screening of siRNA Sequences

Three siRNAs targeting human *PTEN* were designed and screened (Shanghai GenePharma Co., Ltd., Shanghai, China). The siRNA sequences are as follows (**Table 1**):

**Table 1:** PTEN-siRNA sequence.

siRNA sequence number	Sequences	
PTEN-siRNA-65 (S1)	Sense:	5’-GGAGGAUUAUUCGUCUUCUTT-3’
	Antisense:	5’-AGAAGACGAAUAAUCCUCCTT-3’
PTEN-siRNA-1156 (S2)	Sense:	5’-GAAGGCGUAUACAGGAACATT-3’
	Antisense:	5’-UGUUCCUGUAUACGCCUUCTT-3’
PTEN-siRNA-1361 (S3)	Sense:	5’-GGCUAAGUGAAGAUGACAATT-3’
	Antisense:	5’-UUGUCAUCUUCACUUAGCCTT-3’
Negative control siRNA (NC)	Sense:	5’-UUCUCCGAACGUGUCACGUTT-3’
	Antisense:	5’-ACGUGACACGUUCGGAGAATT-3’

The siRNA was mixed with the basal medium in one tube, while Lipofectamine 2000 transfection reagent (Thermo Fisher Scientific, Waltham, MA, USA) was mixed with the basal medium in another tube. After standing for 5 min, the solutions in the two tubes were mixed. The cells were incubated for another 20 min to form a transfection complex and added to the EC9706 cell culture medium. The final concentration of siRNA was 16.7 nM. After 6 h, an inverted fluorescence microscope (BX5; Olympus) was used to observe the transfection efficiency. After 48 h, the gene silencing effect was detected at the mRNA and protein levels.

### Transfection of Cells With siRNA and Overexpression Plasmid

The PTEN overexpression plasmid (PTEN-pEX1) was designed and synthetized by Shanghai GenePharma Co.). The transfected cells were grouped as follows: control group (NC), blank plasmid group (pEX1-NC), PTEN overexpression plasmid group (pEX1), scrambled siRNA group (siRNA-NC), and PTEN-siRNA group (siRNA). The Lipofectamine 2000 transfection reagent was used to transfect the plasmid or siRNA into EC9706 cells. After 48 h, the effect of gene silencing was tested at the mRNA and protein levels. The esophageal cancer cell supernatant was collected from the NC, pEX1, and siRNA groups as the macrophage conditioning medium.

### Induction of M1 TAMs

Phosphomolybdic acid (PMA) (100 ng/mL) was added to the THP-1 cell culture medium and cultured for 48 h to induce M0 type macrophages (M0 TAMs). Then, lipopolysaccharide (LPS) (100 ng/mL) and interferon (IFN)-γ (50 ng/mL) were added to the M0 TAM medium and cultured for 48 h, following which cells were induced to M1 TAMs. Interleukin-4 (100 ng/mL) was added to the M0 TAM medium, and after 48 h of culture, cells were induced to M2 TAMs. After culturing the M1 TAMs with the three groups of macrophage conditioning medium (mentioned in the previous section) for 48 h, the morphology of each group of macrophages was observed under a microscope. The macrophage supernatants of the NC, pEX1, and siRNA groups were collected as tumor-associated vascular endothelial cell (TEC)-conditioned medium.

### TEC Induction and Cultivation in Conditioned Medium

The EC9706 cell supernatant was collected, centrifuged, and filtered as a conditioned medium to cultivate VECs. After 48 h of culture, the VECs were induced to become TECs. The functions of the induced TECs were then verified. Three groups of TEC-conditioned medium were used for culture: NC, pEX1, and siRNA. NC group: the EC9706 cell supernatant was cultured with M1 TAMs, following which the macrophage supernatant was collected as the conditioned medium; pEX1 group: the supernatant of PTEN-pEX1-transfected EC9706 cells was cultured with M1 TAMs, following which the macrophage supernatant was collected as the conditioned medium; siRNA group: the supernatant of the PTEN-siRNA-transfected EC9706 cells was cultured with M1 TAMs, following which the macrophage supernatant was collected as the conditioned medium.

### Immunocytochemistry

Macrophages from each group were fixed with 4% paraformaldehyde, and the SP linkage method was used for immunocytochemical staining using the SP kit (Zhongshan Golden Bridge Biotechnology Co.). Endogenous peroxidase was inactivated with 3% H_2_O_2_ for 20 min, after which sections were blocked with 10% normal goat serum and incubated with primary antibodies overnight at 4°C. Biotin-labeled goat anti-rabbit/mouse IgG was then incubated with cells for 10 min, followed by horseradish peroxidase-labeled streptavidin for another 10 min. Between each incubation step, cells were washed with PBS three times, 3 min each. The following antibodies were used to detect the antigen: anti-CD16 rabbit monoclonal antibody (1:200; ab246222; Abcam) and anti-CD163 rabbit polyclonal antibody (1:200; bs-2527R; Bioss, Beijing, China). The DAB chromogenic solution was added dropwise and incubated for 1 min, following which the samples were counterstained with hematoxylin and mounted on the film after dehydration. CD16 and CD163 expression in the macrophages in each group were observed using a microscope.

### Enzyme-Linked Immunosorbent Assay (ELISA)

The human VEGF ELISA kit (bsk11024; Bioss) was used to detect VEGF content in various cell supernatants. The absorbance (OD value) of each well was measured at 450 nm, and a VEGF standard curve was generated based on the known standard concentration and OD value. A standard curve was used to calculate the VEGF content in each sample.

### Quantitative Reverse Transcription-Polymerase Chain Reaction (qRT-PCR)

Total RNA was extracted using the TRIzol reagent, and its concentration was determined using a microspectrophotometer (Thermo Fisher Scientific). RNA was reverse transcribed into cDNA using the PrimeScript™ RT reagent kit (RR047A; Takara Bio, Japan) under the following conditions: 37°C for 15 min, 85°C for 5 s, and standby at 4°C. PCR was performed using the TB Green™ Premix Ex Taq II reagent kit (RR820; Takara Bio, Japan). The reaction conditions were as follows: pre-denaturation at 95°C for 30 s; 40 cycles of 95°C for 5 s; 60°C for 34 s; dissolution curve: 95°C, 15 s; 60°C, 1 min; 95°C, 15 s. The amplification and melting curves were obtained after completion of the reaction and the expression of target gene was analyzed using the 2^-∆∆CT^ relative quantitative method. The sequences of the PCR primers are as follows (**Table 2**):

**Table 2:** Primer sequences of PTEN, PI3K, and AKt.

PCR primers	Sequences	
PTEN	Forward:	5’-TGGATTCGACTTAGACTTGACCT-3’
	Reverse:	5’-GGTGGGTTATGGTCTTCAAAAGG-3’
PI3K	Forward:	5’-TATTTGGACTTTGCGACAAGACT-3’
	Reverse:	5’-TCGAACGTACTGGTCTGGATAG-3’
AKt	Forward:	5’-AGCGACGTGGCTATTGTGAAG-3’
	Reverse:	5’-GCCATCATTCTTGAGGAGGAAGT-3’
GAPDH	Forward:	5’-CTTAGCACCCCTGGCCAAG-3’
	Reverse:	5’-GATGTTCTGGAGAGCCCCG-3’

### Western Blotting

Radioimmunoprecipitation assay lysis buffer (Solarbio Science & Technology Co.) containing 1% phenylmethylsulfonyl fluoride was used to extract total proteins from the cells. Protein concentration was determined using a bicinchoninic acid kit (Thermo Fisher Scientific). Equal amounts of proteins were electrophoresed on a 10% sodium dodecyl sulfate-polyacrylamide gel and the proteins were transferred to a polyvinylidene fluoride membrane. A 5% bovine serum albumin solution (Solarbio Science & Technology Co.) was used to block non-specific protein sites, following which the membrane was washed with Tris-buffered saline-Tween 20 (TBST) and incubated overnight with the primary antibody at 4°C. The following antibodies were used to detect the antigen: anti-PTEN rabbit monoclonal antibody (1:1000; ab267787; Abcam), anti-PI3K mouse monoclonal antibody (1:5000; 67121-1; Proteintech Group), anti-phospho-AkT (P-AKT) rabbit monoclonal antibody (1:1000; 4691; Cell Signaling Technology), and anti-GAPDH rabbit polyclonal antibody (1:2500; ab9485; Abcam). The next day, the membranes were incubated with horseradish peroxidase-labeled goat anti-rabbit/mouse IgG (1:10000; ab6721/ab6728; Abcam). After washing with TBST, an appropriate amount of ECL chemiluminescence solution (Thermo Fisher Scientific) was added dropwise, and images were captured using a multifunctional gel imaging system (Syngene, Cambridge, UK). PBS was used as the control instead of the primary antibody in each experiment. After the experiment, the intensity of bands in each group was calculated using the ImageJ software (National Institutes of Health, Bethesda, Maryland, USA), and the relative protein content of each group of cells was obtained.

### Cell Proliferation Assay

Cells (3 × 10^3^ cells/well) were seeded in a 96-well plate, and 10 μL CCK-8 detection reagent (Solarbio Science & Technology Co.) was added to each well while avoiding the formation of air bubbles and incubated in the dark. Three wells were used for each group. The absorbance (OD value) of each well was measured at 450 nm and cell proliferation ability was measured after 24, 48, and 72 h.

### Cell Migration Assay

Three horizontal lines were drawn on the back of a 6-well plate using a marker to indicate the position. In total, 5 × 10^5^ cells were inoculated in each well of a 6-well plate. After the culture was 95% confluent, a 200 μL pipette tip was used to scratch vertical lines on the plate surface. Images were captured at 0, 12, and 24 h. The ImageJ software was used to analyze the distance migrated.

### Cell Invasion Assay

Matrigel (BD Biosciences, Franklin Lakes, NJ, USA) and basal DMEM were mixed in a 1:4 ratio, and 50 μL of the diluted Matrigel was evenly spread on the upper layer of each small 24-well plate chamber (BD Biosciences), avoiding the formation of air bubbles. After Matrigel solidified, 200 μL cell suspension (3 × 10^4^ cells) was added to the upper layer of the small chamber, and 500 μL complete DMEM containing 20% serum was added to the lower layer of the small chamber. After culturing in a cell incubator for 16 h, the cells were fixed in 4% paraformaldehyde for 20 min and stained with 0.1% crystal violet solution for 20 min. After air-drying, five fields of view were randomly captured in each chamber using a microscope. The ImageJ software was used to analyze the number of cells passing through the chamber.

### Tube Formation Assay

The Matrigel and serum-free DMEM were mixed in a 1:2 ratio, and 200 µL of the diluted Matrigel was added to each well of a 24-well plate while avoiding air bubbles. After the Matrigel had solidified, 10^5^ cells were seeded per well in a 24-well plate, and the ells were photographed and recorded under a microscope at 0, 2, 4, 6, 8, 10, and 12 h. The ImageJ software was used to determine the total length of the loop branches.

### Statistical Analysis

The results were obtained through three independent repeated experiments, each group of samples was repeated three times. The SPSS21.0 software (IBM, Chicago, IL, USA) was used for statistical analysis. The measurement data were expressed as average ± standard deviation. Differences between two groups were compared using the LSD-t test, while comparisons among multiple groups were performed using one-way analysis of variance. When data did not conform to the normal distribution or the variance was uneven, a non-parametric test (Kruskal–Wallis test) was used for comparison between groups. The χ^2^ test or Fisher’s exact probability method was used for comparison of count data and the correlation between each index and clinicopathological parameters, and the Spearman’s method was used for correlation analysis. The test level α = 0.05 and *p* < 0.05 was considered statistically significant.

## Results

### Differences in TAM Infiltration, MVD, and PTEN/PI3K/AKT Expression in ESCC and Adjacent Normal Tissues

**Figure 1:**
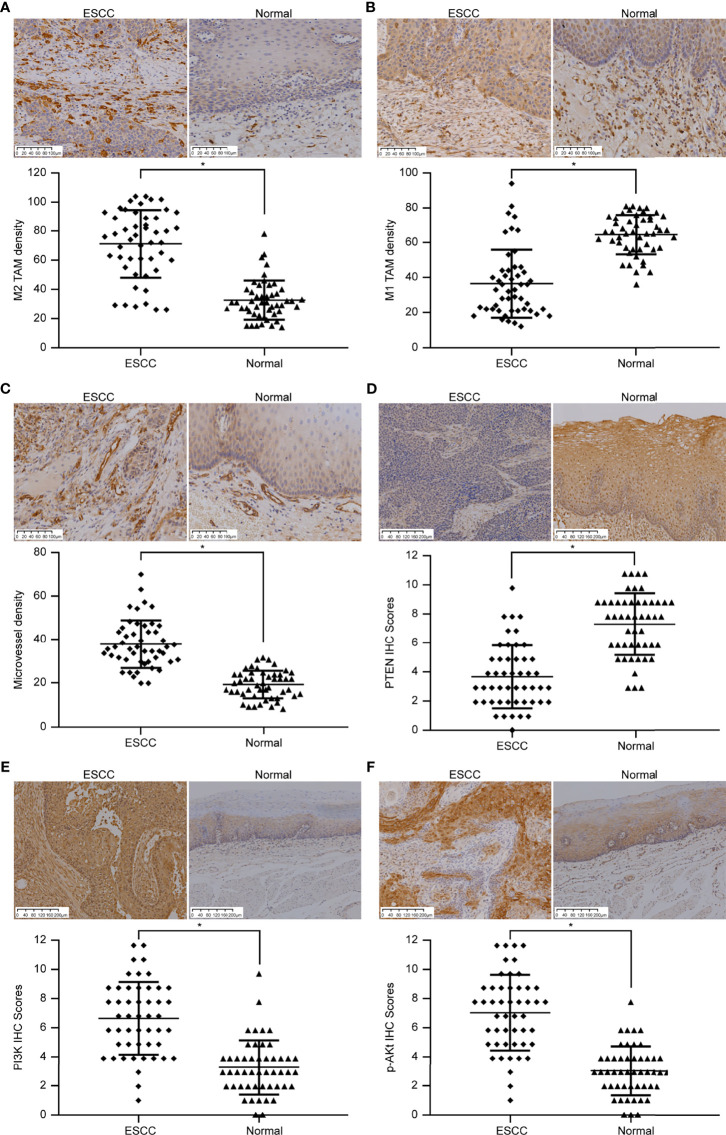
Immunohistochemical images showing the differences in TAM infiltration, MVD value, and PTEN/PI3K/AKT protein expression in ESCC and adjacent normal tissues. **(A)** Image showing CD163-labeled M2 TAMs. The amount of M2 TAM infiltration in ESCC is higher than that in the normal tissues adjacent to the cancer (SP method, 200×). **(B)** Image showing CD16-labeled M1 TAMs. The extent of M1 TAM infiltration in ESCC is lesser than that in normal tissues adjacent to the cancer (SP method, 200×). **(C)** Image showing CD31-labeled vascular endothelial cells. The MVD value in ESCC is higher than that in normal tissues adjacent to the cancer (SP method, 200×). **(D)** Image showing PTEN staining. PTEN expression score in ESCC is lower than that in normal tissues adjacent to the cancer (SP method, 100×). **(E)** Image showing PI3K staining. PI3K expression score in ESCC is higher than that in normal tissues adjacent to the cancer (SP method, 100×). **(F)** Image showing p-AKT staining. p-AKT expression score in ESCC is higher than that in normal tissues adjacent to the cancer (SP method, 100×). *p < 0.05.

CD163 was used to label M2 TAMs (**Figure 1A**). We observed that M2 TAMs were scattered in ESCC tumor nests and that a large number of M2 TAMs had infiltrated the tumor stroma, the number of infiltrating cells being 71.25 ± 23.29/high-power field (HP). The number of infiltrated M2 TAMs in the adjacent tissues was significantly lower than that in the tumor tissues, and the number of infiltrations was 32.57 ± 13.48/HP, which was mainly distributed in the mucosal layer. The number of CD16-labeled infiltrated M1 TAMs in ESCC was 36.51 ± 19.43/HP (**Figure 1B**), while that in adjacent tissues was 64.53 ± 11.34/HP. The number of infiltrated M1 TAMs in adjacent tissues was significantly higher than that in ESCC tissues. The differences in the extent of invasion of M1 and M2 TAMs in ESCC and adjacent tissues was significant (*p* < 0.05).

CD31 was used to label VECs (**Figure 1C**). A large number of new blood vessels were observed in the tumor stroma in ESCC tissues, and the MVD value was 38.33 ± 11.18 cells/HP. In the adjacent tissues, moderate number of microvessels were distributed under the squamous epithelium, and the MVD value was 19.39 ± 6.44/HP. MVD values differed significantly between ESCC and adjacent tissues (*p <* 0.05).

PTEN expression was low in ESCC (**Figure 1D**). Only 16 of 49 cases of ESCC showed high expression (positive rate 32.66%), whereas 45 of 49 cases of adjacent tissues showed high expression (positive rate 91.83%). PTEN level in ESCC and adjacent tissues differed significantly (*p* < 0.05). PI3K was highly expressed in ESCC (**Figure 1E**); 38 of 49 ESCCs highly expressed PI3K (positive rate 77.56%), whereas 10 of 49 cases in adjacent tissues showed high levels of PI3K (positive rate 20.40%). p-AKT and PI3K levels were similar in ESCC tissues (**Figure 1F**). Among 49 cases of ESCC, 41 cases expressed high levels of p-AKT (positive rate 83.67%), while 9 of 49 cases of adjacent tissues highly expressed p-AKT (positive rate 18.37%). The expression of PI3K and p-AKT in ESCC and adjacent tissues differed significantly (*p* < 0.05).

### Correlation Between TAM Infiltration, MVD Value, PTEN/PI3K/p-AKT Expression, and Clinicopathological Parameters in ESCC

We obtained the extent of M1/M2 TAM infiltration, MVD value, and PTEN/PI3K/p-AKT protein expression score in ESCC using immunohistochemical experiments, and then analyzed their correlation with clinicopathological parameters using the chi-square test (**Table 3**). Clinicopathological parameters included sex, age, tumor diameter, histology grade, depth of invasion, lymph node status, and pathological stage. The results showed that the number of TAMs and PI3K level were related to the depth of tumor invasion, lymph node metastasis, and pathological stage (*p* < 0.05), but not related to other parameters (*p >* 0.05). MVD value and PTEN/p-AKT protein level were related to tumor diameter, depth of invasion, lymph node metastasis, and pathological stage (*p <* 0.05), but were not related to the other parameters (*p >* 0.05).

**Table 3:** Correlation between the extent of TAM infiltration, MVD value, PTEN/PI3K/p-AKT expression, and clinicopathological parameters.

Variable	Cases(N)	Number of M1 TAMs				Number of M2 TAMs				Number of MVD				Expression of PTEN				Expression of PI3K				Expression of p-AKT			
		High	Low	χ^2^	*p*	High	Low	χ^2^	*p*	High	Low	χ^2^	*p*	High	Low	χ^2^	*p*	High	Low	χ^2^	*p*	High	Low	χ^2^	*p*
Gender																										
Male	32	25	7	2.079	0.149	5	27	0.033	0.855	26	6	0.397	0.529	10	22	0.083	0.774	23	9	1.707	0.191	25	7	2.079	0.149
Female	17	16	1			3	14			15	2			6	11			15	2			16	1		
Age (years)																										
<60	13	10	3	0.590	0.442	4	9	2.702	0.100	10	3	0.590	0.442	6	7	1.467	0.226	11	2	0.507	0.476	11	2	0.011	0.915
≥60	36	31)	5			4	32			31	5			10	26			27	9			30	6		
Tumor diameter (cm)																									
<3	17	12	5	3.263	0.071	5	12	3.263	0.071	11	6	6.855	0.009^*^	10	7	8.107	0.004^*^	11	6	2.467	0.116	11	6	6.855	0.009^*^
≥3	32	29	3			3	29			30	2			6	26			27	5			30	2		
Histology grade																									
G1–G2	36	29	7	0.966	0.326	7	29	0.966	0.326	30	6	0.011	0.915	13	23	0.738	0.390	27	9	0.507	0.476	30	6	0.011	0.915
G3	13	12	1			1	12			11	2			3	10			11	2			11	2		
Depth of invasion																									
T1–T2	24	16	8	9.959	0.002^*^	7	17	5.677	0.017^*^	17	7	5.677	0.017^*^	13	11	9.900	0.002^*^	14	10	9.979	0.002^*^	16	8	9.959	0.002^*^
T3–T4	25	25	0			1	24			24	1			3	22			24	1			25	0		
Lymph node status																									
N^−^	32	24	8	5.079	0.038^*^	8	24	5.079	0.038^*^	24	8	5.079	0.038^*^	15	17	8.483	0.004^*^	22	10	4.104	0.043^*^	24	8	5.079	0.038^*^
N^+^	17	17	0			0	17			17	0			1	16			16	1			17	0		
Pathological stage																									
I-II	32	24	8	5.079	0.038^*^	8	24	5.079	0.038^*^	24	8	5.079	0.038^*^	15	17	8.483	0.004^*^	22	10	4.104	0.043^*^	24	8	5.079	0.038^*^
III-IV	17	17	0			0	17			17	0			1	16			16	1			17	0		

N^+^, Lymph node metastasis; N^−^, No lymph node metastasis; MVD, Microvessel density. ^*^p < 0.05.

### Correlation Between PTEN/PI3K/AKT Expression, Extent of TAM Infiltration, and MVD Value in ESCC

**Figure 2:**
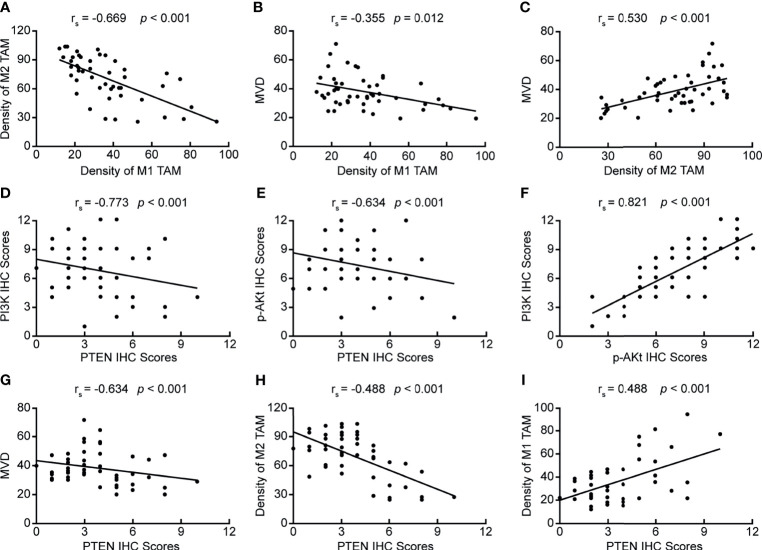
Correlation between PTEN/PI3K/AKT expression, the number of infiltrating TAMs, and MVD value. **(A)** Correlation between the numbers of infiltrating M2 TAMs and M1 TAMs; **(B)** Correlation between the numbers of infiltrating M1 TAMs and MVD value; **(C)** Correlation between the number of infiltrating M2 TAMs and MVD value; **(D)** Correlation between PI3K and PTEN protein levels; **(E)** Correlation between p-AKT and PTEN levels; **(F)** Correlation between PI3K and p-AKT levels; **(G)** Correlation between PTEN expression and MVD value; **(H)** Correlation between PTEN expression and the number of infiltrating M2 TAMs; **(I)** Correlation between PTEN expression and the number of infiltrating M1 TAMs. *p <* 0.05 was considered to be statistically significant.

By analyzing the correlation between PTEN/PI3K/AKT protein expression, the number of infiltrated TAMs, MVD value, and clinicopathological data, we found that all these indicators were related to the depth of tumor invasion, lymph node metastasis, and pathological staging, suggesting that these indicators may be related. Therefore, we further analyzed the correlation between PTEN/PI3K/AKT protein expression, the number of infiltrated TAMs, and MVD values. First, we analyzed the correlation between the number of infiltrated TAMs and MVD value, and found negative correlation between the number of infiltrated M1 TAMs and M2 TAMs (**Figure 2A**) (rs = −0.669, *p* < 0.05), and the number of infiltrated M1 TAMs and MVD value (**Figure 2B**) (rs = −0.355, *p* = 0.012). The extent of M2 TAM infiltration correlated positively with the MVD value (**Figure 2C**) (rs = 0.530, *p* < 0.05). We then analyzed the correlation between the expression of the three key proteins of the PI3K/PTEN/AKT pathway. PTEN expression correlated negatively with PI3K and p-AKT protein levels (**Figures 2D, E**) (rs = −0.773, *p* < 0.05; rs = −0.634, *p* < 0.05); PI3K and p-AKT protein levels showed positive correlation (**Figure 2F**) (rs = 0.821, *p* < 0.05), indicating that PTEN is an important negative regulator of the PI3K/AKT signaling pathway. Finally, we analyzed the correlation between the expression of PTEN, extent of TAM infiltration, and MVD value, and found that PTEN level correlated negatively with the MVD value and extent of M2 TAM infiltration (**Figures 2G, H**) (rs =−0.634, *p* < 0.05; rs =−0.488, *p* < 0.05), while it correlated positively with the number of infiltrated M1 TAMs (**Figure 2I**) (rs = 0.488, *p* < 0.05). Therefore, we speculated that tumor cells in ESCC activate the PI3K/AKT pathway by down-regulating PTEN, inducing the polarization of M2 TAMs in the tumor microenvironment, and ultimately promoting the formation of microvessels.

### Silencing or Up-Regulation of PTEN in EC9706 Cells

**Figure 3:**
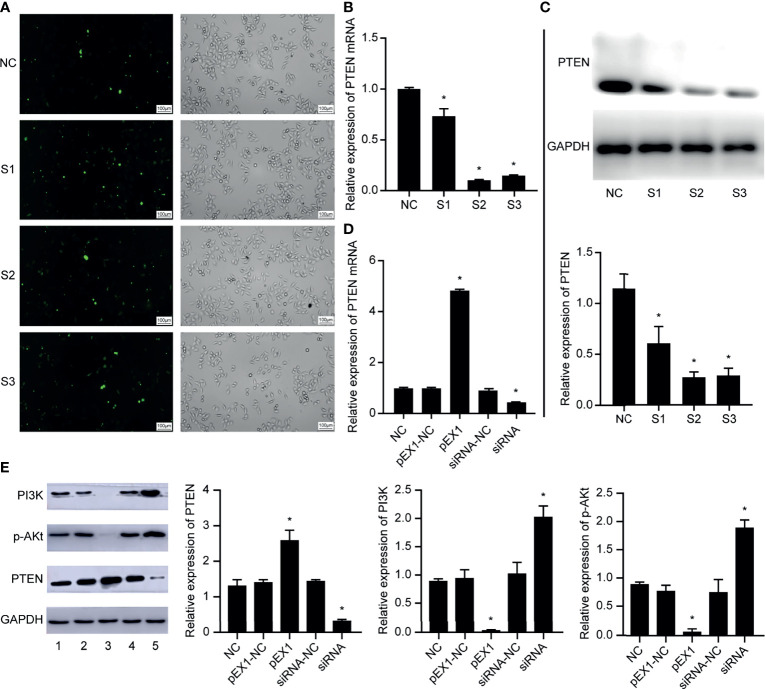
Validation of EC9706 cell transfection experiment results. **(A)** Fluorescence microscope image of esophageal cancer cells transfected with fluorescently-labeled siRNA (200×); **(B)** Relative expression of PTEN mRNA in each group of EC9706 cells transfected with siRNA; **(C)** Relative expression of PTEN in each group of esophageal cancer cells transfected with siRNA; **(D)** Relative expression of PTEN mRNA in EC9706 cells transfected with siRNA and pEX1 (siRNA group: S2 sequence); **(E)** Relative expression of PTEN/PI3K/p-AKT in EC9706 cells transfected with siRNA and pEX1 (1: NC group; 2: pEX1-NC group; 3: pEX1 group; 4: siRNA-NC group; 5: siRNA group) (siRNA group: S2 sequence). *Compared with NC group, *p* < 0.05.

We designed and synthesized three siRNA sequences targeting *PTEN* and transfected them in EC9706 cells. After 6 h of transfection, the transfection efficiency of fluorescently-labeled FAM (Carboxyfluorescein) siRNA was observed using an inverted fluorescence microscope (**Figure 3A**). The results showed that the EC9706 cells in the NC, S1, S2, and S3 groups showed fluorescent signals, and that the transfection efficiencies of the four groups were similar. The silencing efficiency of *PTEN* in the four groups of EC9706 cells was detected using qRT-PCR (**Figure 3B**); *PTEN* silencing was best in the S2 group. PTEN protein levels in the four groups of transfected EC9706 cells was detected using western blotting (**Figure 3C**). PTEN content in the S2 group was the lowest, which was consistent with the results of qRT-PCR analysis, indicating that PTEN was silenced maximally by S2, which was used in subsequent experiments. Therefore, the S2 sequence is used in all siRNAs in subsequent experiments.

Then, the EC9706 cells were transfected with scrambled siRNA, S2 siRNA, blank plasmids, and PTEN overexpression plasmids. PTEN expression in each group was detected using qRT-PCR (**Figure 3D**). PTEN expression did not differ in the NC, pEX1-NC, and siRNA-NC groups (*p >* 0.05). The expression of PTEN in the pEX1 group was significantly higher than that in the siRNA group, where PTEN was significantly silenced (*p* < 0.05). PTEN and PI3K/p-AKT expression in each group was detected using western blotting (**Figure 3E**). PTEN, PI3K, and p-AKT expression did not differ in the NC, pEX1-NC, and siRNA-NC groups (*p >* 0.05). PTEN was up-regulated in the pEX1 group, whereas PI3K and AKT were significantly suppressed (*p* < 0.05); opposite results were obtained in the siRNA group, where PTEN level decreased, whereas PI3K/AKT levels increased (*p* < 0.05). This suggested that PTEN was a key negative regulator of the PI3K/AKT signaling pathway.

### Macrophage Conditioning Medium Induced Polarization of M1 TAMs

**Figure 4:**
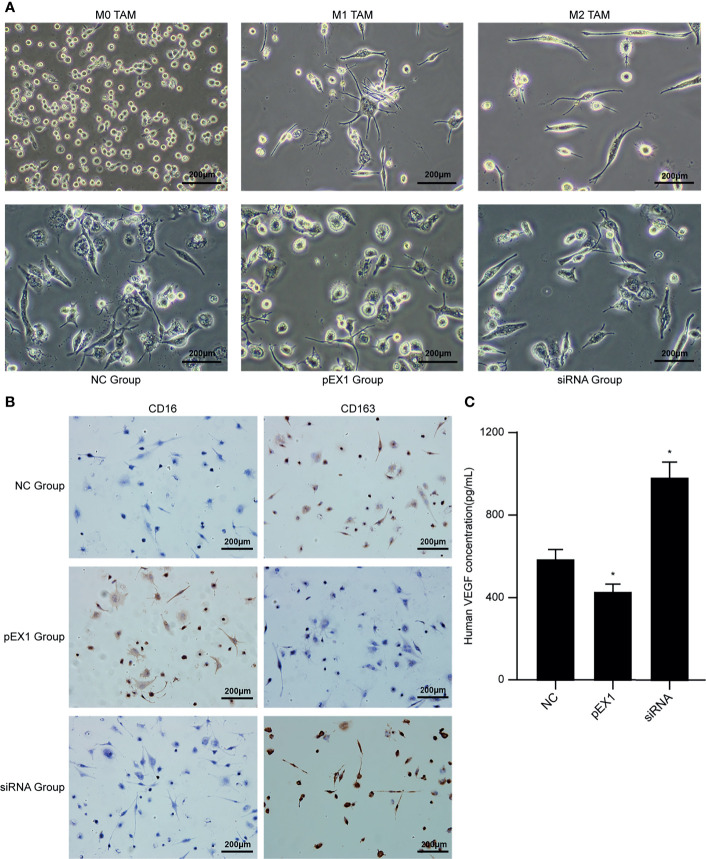
Conditioned medium induced polarization of M1 TAMs. **(A)** Microscopic observation of M0/M1/M2 TAMs and induced macrophages in each group (100×); **(B)** Immunocytochemical method for phenotypic identification of macrophages in each group (100×); **(C)** VEGF content in the supernatant of macrophages in each group. (siRNA group: S2 sequence). *p < 0.05 compared to the NC group.

THP-1 is an elliptical suspension cell; M0 TAMs grow adherently, albeit with elliptical morphology; M1 TAMs are irregular in shape with multiple radial antennae, while M2 TAMs become fusiform and grow elongated antennae on both sides. The above morphologies were used for detecting the macrophage populations after treatment with conditioning medium. After culturing M1 TAMs with macrophage conditioning medium for 48 h (**Figure 4A**), we observed that the pEX1 group was predominantly composed of M1 TAMs, the M1 TAMs in siRNA group were polarized as M2, and the NC group consisted mostly of M2 TAMs and some M1 TAMs. The phenotype of the M1 TAMs induced by the conditioning medium of each group was identified using immunocytochemistry (**Figure 4B**). The results showed that the macrophages of the NC group did not express CD16, but weakly expressed CD163. The macrophages in the pEX1 group showed high expression of CD16 but did not express CD163. The macrophages in the siRNA group were negative for CD16 and positive for CD163, and the expression intensity was significantly higher than that in the NC group. Observation of macrophage morphology under the microscope indicated that the phenotype of the macrophages in the NC group was similar to that of the M2 type, while the macrophages in the pEX1 group were still of the M1 type and those in the siRNA group were completely polarized to the M2 type.

The supernatants of the macrophages were collected from each group to detect VEGF content (**Figure 4C**). The VEGF level in the cell supernatant of the pEX1 group was the lowest (approximately 432 pg/mL), while that in the cell supernatant of the siRNA group was the highest (approximately 986 pg/mL), and the VEGF content in the cell supernatant of the NC group was approximately 594 pg/mL. The difference between the two experimental groups and the NC group was significant (*p* ≤ 0.05). VEGF is the most important positive regulator of angiogenesis. Macrophages in the siRNA group tended to be of the M2 type, suggesting that VEGF secretion increased after M1 TAMs were polarized to the M2 type, which promoted tumor neovascularization.

### M1 TAMs Induced Polarization to M2 TAMs *via* the PI3K/AKT Signaling Pathway

**Figure 5:**
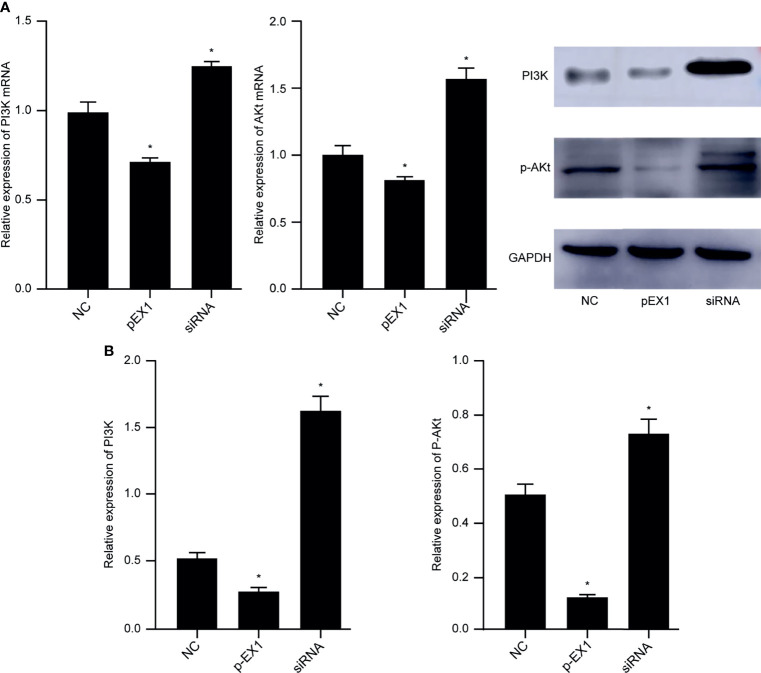
PI3K/AKT signaling pathway in macrophages of each group. **(A)** Expression of PI3K and AKT genes in macrophages of each group; **(B)** PI3K and p-AKT protein levels in macrophages of each group. (siRNA group: S2 sequence). *p < 0.05 compared to the NC group.

The macrophages induced by polarization in the NC, pEX1, and siRNA groups were collected, and RNA and protein were extracted to verify the activation state of the PI3K/AKT signaling pathway. The results showed that compared to that in the NC group, the PI3K/AKT signaling pathway was activated and the genes encoding PI3K and AKT were highly expressed in the macrophages of the siRNA group. In contrast, the expression of PI3K and AKT genes in the pEX1 group was significantly lower than that in the NC group (*p* < 0.05) (**Figure 5A**). The protein expression in the macrophages of each group was consistent with the gene expression (**Figure 5B**). PI3K and p-AKT were up-regulated in the siRNA group, indicating activation of the PI3K/AKT signaling pathway. The PI3K and p-AKT levels in the macrophages of the pEX1 group were lower than those in the NC group (*p* < 0.05). Down-regulation of PTEN in esophageal cancer cells can activate the PI3K/AKT signaling pathway in TAMs *via* the tumor microenvironment and promote the polarization of M1 TAMs to M2 type.

### Culturing in Conditioned Medium Induced VECs to TECs

**Figure 6:**
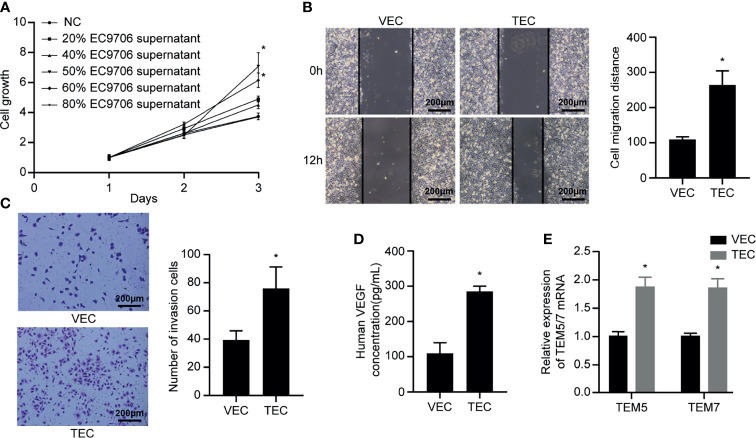
VECs cultured in conditioned medium to induce TECs. **(A)** Proliferation ability of TECs induced by different concentrations of conditioned medium; **(B)** Migration ability of VECs and TECs (100×); **(C)** Invasion ability of VECs and TECs (100×); **(D)** VEGF content in the supernatant of VECs and TECs; **(E)** Levels of tumor endothelial markers in VECs and TECs. *p < 0.05 compared to VECs.

The supernatant of EC9706 cells was collected and prepared as a conditioned medium to induce VECs to become TECs. The CCK-8 method was used to determine the concentration of the conditioned medium optimal for induction (**Figure 6A**). The TEC proliferation ability induced by the conditioned medium containing 50% EC9706 cell supernatant was the strongest (*p* < 0.05). This indicated that the 50% EC9706 cell supernatant was optimal for TEC induction and was used in subsequent experiments to induce TEC.

The scratch experiment showed that the TECs migrated farther at the same time (**Figure 6B**) (*p* < 0.05). Transwell invasion experiments revealed that the number of cells passing through the chamber in the TEC group was significantly higher than that in the VEC group at the same time (**Figure 6C**) (*p* < 0.05). The results of ELISA showed that the VEGF content in the TEC cell supernatant was approximately 280 pg/mL (**Figure 6D**), while the VEGF concentration in the VEC cell supernatant was only 107 pg/mL, and the difference was statistically significant (*p* < 0.05). The mRNA levels of the tumor-related endothelial cell markers, TEM5 and TEM7, in VECs and TECs were determined (**Figure 6E**). The results showed that TEM5 and TEM7 were both highly expressed in TEC cells, and that the mRNA expression was about 1.8 times higher than that of VECs (*p* < 0.05). This suggested that the phenotype of TECs obtained from conditional culture-induced VECs was similar to that of tumor-associated endothelial cells. The ability of TECs to secrete VEGF was thrice that of VECs, indicating that they promoted angiogenesis more strongly than VECs, and possessed improved migration and invasion capabilities.

### Effect of Inducing Polarization of M1 TAMs on TEC Function

**Figure 7:**
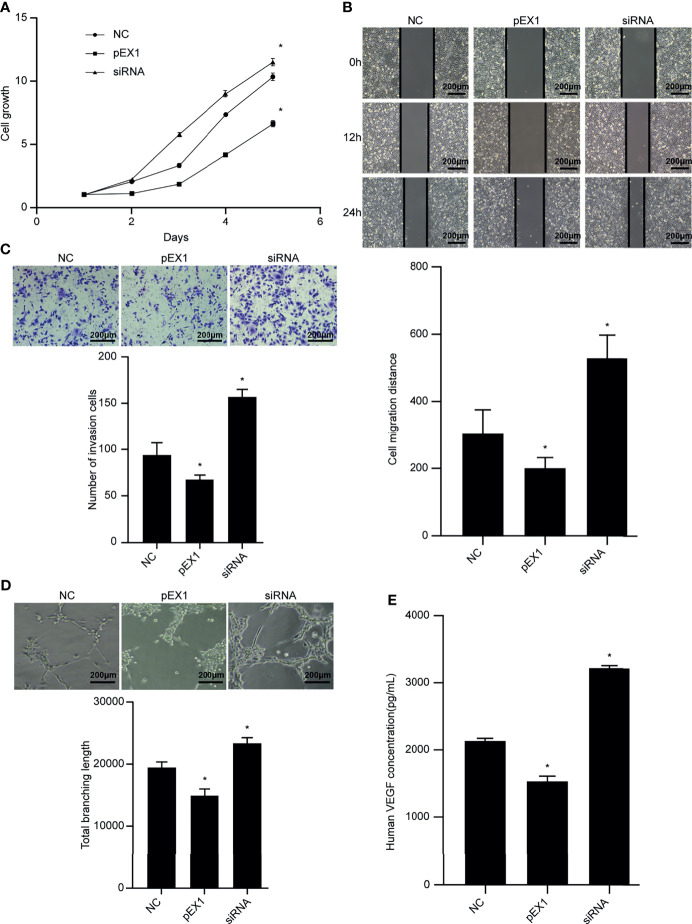
Biological functions of TECs in each group. **(A)** Cell proliferation ability after TECs were cultured in different conditioned media; **(B)** Cell migration ability after TECs were cultured in different conditioned media (100×); **(C)** Cell invasion ability after TECs were cultured in different conditioned media (100×); **(D)** Tube-forming ability after TECs were cultured in different conditioned media (100×); **(E)** VEGF secretion after culturing TECs in different conditioned media. (siRNA group: S2 sequence). *p < 0.05 compared to the NC group.

After culturing the TECs in TEC-conditioned medium for 48 h, the CCK-8 reagent was used to detect the proliferation ability of TECs in each group (**Figure 7A**). In the pEX1 group, the cell growth rate increased significantly from the second day, whereas cell proliferation in the siRNA group was the slowest. The scratch experiment revealed that the TECs in the siRNA group migrated the most (**Figure 7B**), whereas those in the pEX1 group migrated the least. The migration distances of the two experimental groups differed from that of the NC group (*p* < 0.05). The Transwell invasion experiment revealed that the number of TECs passing through the chamber in the siRNA group was the largest (**Figure 7C**), while the number of cells passing through the chamber in the pEX1 group was only 50% of that in the siRNA group. The difference between the two experimental groups and the NC group was statistically significant (*p* ≤ 0.05). Tube formation experiments were used to detect the tube-forming ability of each group of TECs (**Figure 7D**). The results showed that 8 h after inoculation with the cell suspension, a complete vascular structure was formed in the siRNA group and a small number of tubular structures was formed in the NC group, whereas no structure was formed the NC group. Analysis of the complete tubular structure using the ImageJ software revealed that the total length of the tubular structure branch in the siRNA group was longer than that in the NC group and pEX1 group, and that the total branch length in the pEX1 group was the shortest (*p* < 0.05). The results of ELISA revealed that the VEGF content in the cell supernatant of the pEX1 group was approximately 3053 pg/mL (**Figure 7E**), while that in the cell supernatant of the NC group was 2166 pg/mL. The VEGF concentration in the cell supernatant of the pEX1 group was only 1365 pg/mL, and the VEGF secretion capacity of the cells in the siRNA group was three times that of the cells in the pEX1 group (*p* < 0.05). This suggested that silencing of PTEN in EC9706 cells can induce macrophage polarization to the M2 type, promote proliferation, migration, invasion, tube formation, and secretion of VEGF in tumor-associated endothelial cells, and ultimately contribute to tumor angiogenesis.

## Discussion

Owing to its highly metastatic nature, vascular and lymphatic metastasis of esophageal tumor cells occurs even in patients who do not show any obvious clinical symptoms in the early stage of the disease (13, 14). Therefore, in-depth research on the molecular pathology and neovascularization mechanisms of esophageal cancer are urgently required to develop new strategies for early diagnosis and improvement of prognosis.

A series of cytokines and inflammatory mediators, such as interleukin-10, transforming growth factor-beta, and prostaglandin E2, are produced in the tumor microenvironment (15). Various cytokines and inflammatory mediators form a complex TAM regulatory network, which affects the malignant biological behavior of tumor cells by regulating the TAM phenotype (15). In this study, we investigated TAMs in the ESCC microenvironment and found that they are mainly distributed in the tumor stroma, with only a small diffuse distribution in the tumor nest, and that the extent of M2 TAM infiltration in the ESCC tissue was significantly higher than that in the normal tissue adjacent to the cancer. The reverse was observed for M1 TAMs. High-density M1 TAM tumors often indicate low malignancy, shallow invasion depth, and absence of lymph node metastasis. Tumors with a high density of M2 TAMs at the margins of the tumor stroma, cancer nests, and muscular invasion have higher degree of malignancy. The amount of TAM infiltration in ESCC tissue is similar to that in other solid malignant tumors, such as breast cancer, gastric cancer, and lung cancer. A large amount of M2 TAM infiltration was observed in tumor tissue (16–18). Sugimura et al. (19) analyzed 210 ESCC tissue samples and found that a large number of CD163-positive TAMs infiltrated the tumor tissues, which was related to the depth of tumor invasion, lymph node metastasis, and vascular metastasis. Shigeoka et al. (20) studied 70 patients with ESCC and found that the number of M2 TAMs in esophageal cancer tissue was significantly higher than that in adjacent tissues. Survival analysis showed that the extent of M2 TAM infiltration correlated negatively with patient survival. At present, reports on the distribution of infiltrated M1 TAMs in ESCC and its relationship with clinicopathological parameters are lacking. However, findings similar to those of this study have been reported for ovarian cancer and hepatocellular carcinoma. Patients with ovarian cancer and high density of M1 TAMs have less tumor invasion and metastasis, longer overall survival, and progression-free survival; liver cancer characterized by low density of M1 TAMs is more aggressive, with more lymph node metastases, and TNM pathological staging is poor (21, 22).

Further analysis of the relationship between the extent of TAM infiltration and MVD value revealed that M1 TAMs can interfere with tumor microvascular formation and effectively inhibit tumor progression, while M2 TAMs can promote the malignant biological behavior of tumors. Both *in vitro* tumor cell experiments and *in vivo* animal experiments have confirmed that M2 TAMs can promote tumor growth and metastasis by inducing angiogenesis (23, 24).

The PTEN protein on the plasma membrane mainly acts as a lipid phosphatase, which can convert phosphatidylinositol 3,4,5 triphosphate (PIP3) into phosphatidylinositol 4,5-bisphosphate (PIP2). The activation of PI3K is cancelled, thereby blocking the initiation of the PI3K pathway, and the signaling effect of downstream protein kinase B (AKT) is inhibited. Therefore PTEN can inhibit the growth and metastasis of tumors (25). PTEN is also expressed in the cytoplasm and nucleus, and plays a role in regulating the cell cycle and maintaining the stability of the genome (26). The chromosomal region of PTEN has abnormalities and mutations in various malignant tumors. Loss of PTEN function often promotes tumor malignancy (6, 27). In this study, we found that *PTEN* silencing can significantly up-regulate the protein levels of PI3K and p-AKT, suggesting that PTEN is a key negative regulator of the PI3K/AKT signaling pathway. Furthermore, PTEN is related to the depth of tumor invasion, lymph node metastasis, and pathological staging. Here, we showed that siRNA-mediated targeting of PTEN can enhance the proliferation, migration, and invasion of ESCC cells, and that the malignant phenotype of ESCC is related to the activation of the PI3K/AKT pathway (28–30). PTEN can also regulate the expression of VEGF to inhibit tumor angiogenesis. In ovarian cancer tissues, the expression of PTEN is negatively correlated with clinical stage and tumor differentiation, and it is negatively correlated with the expression level of VEGF (31). Tian et al. (32) found in *in vivo* and *in vitro* experiments that overexpression of PTEN can down-regulate the expression levels of HIF-1α and VEGF, reduce the proliferation and migration of endothelial cells, and reduce angiogenesis in nude mice xenograft tumor.

Studies have shown that abnormal expression of PTEN in tumor cells can affect the number of different immune cell populations in TME (33). TAMs and PTEN are potentially connected. The absence of PTEN in the tumor microenvironment can induce polarization of M2 TAMs and promote tumor metastasis. To further investigate the effect and mechanism of action of PTEN on TAMs in the tumor microenvironment, we performed a correlation analysis between the number of infiltrated TAMs, PTEN expression, and MVD value in ESCC tissues. The results indicated that the two types of TAMs undergo mutual conversion under the influence of inflammatory mediators or cytokines in the tumor microenvironment, and that PTEN may be a regulator of TAM polarization. *In vitro* experiments indicated that silencing of PTEN in esophageal cancer cells can induce polarization of M1 TAMs to the M2 type, and that the mRNA and protein levels of PI3K/AKT in macrophages were significantly increased. Up-regulation of PTEN did not change the phenotype of M1 TAMs. We speculated that down-regulation of PTEN in esophageal cancer cells activates the PI3K/AKT signaling pathway in TAMs *via* the tumor microenvironment, which induces M1 to M2 polarization. Studies have also shown that VEGF secretion increases when M1 TAMs are polarized to the M2 type, which is conducive for the formation of tumor blood vessels. Zhang et al. (34) found that in lung cancer, M2 TAMs can be reconverted to the M1 type *via* a Toll-like receptor 4-mediated pathway. This result confirmed that TAMs are affected by the microenvironment and that M1 and M2 TAMs can transform mutually. Zhao et al. (35) found that down-regulation of PTEN and activation of the PI3K/AKT signaling pathway in colorectal cancer can induce M2 TAM polarization and promote the metastasis of colorectal cancer to the liver. Cheng et al. (36) also proposed that PTEN can regulate M2 TAM polarization by activating the PI3K/AKT/STAT6 signaling pathway. These results support the observations of this study.

The blood vessels in the tumor tissues are composed of TECs. Compared to ordinary endothelial cells, TECs overexpress specific genes and show significantly high proliferation, migration, and tube formation capabilities (37). These phenotypic and functional differences lead to abnormalities in the morphology, structure, and function of tumor blood vessels, such as immature vascular structure, incomplete basement membrane, incomplete connection of endothelial cells, and insufficient blood perfusion. Distortion and deformation of high-permeability neovascularization is a prominent feature of tumor tissue (38, 39). Various cytokines in the tumor microenvironment can stimulate normal endothelial cells to differentiate into TECs, which are further induced to form vascular-like structures.

Our findings provide preliminary evidence for exploring the principles and mechanisms of early metastasis of esophageal cancer. Down-regulation of PTEN expression in esophageal cancer cells can activate the PI3K/AKt signaling pathway in macrophages and induce M1 type macrophages to polarize to M2 type. Enhance the proliferation, migration, invasion and tube formation of TEC cells, and up-regulate the content of VEGF in the tumor microenvironment, thereby promoting tumor angiogenesis. Therefore, it is possible to detect early ESCC by detecting TAM, or to adjust the phenotype of TAM by affecting the expression of PTEN to inhibit tumor angiogenesis, which can be used as a potential direction for early diagnosis and treatment of ESCC. Studies have pointed out that the release of miR-301a-3p in exosomes by pancreatic cancer cells can transform macrophages into M2 type by regulating the expression of PTEN, and promote the formation of lung metastases (7). The release of miR-103a from lung tumor cells can inhibit the activity of PTEN in macrophages, activate the PI3K/AKt pathway to induce the polarization of macrophages to M2 type, and promote tumor metastasis and angiogenesis (8). We speculate that ESCC cells will also release microRNAs, which act on the PI3K/AKt pathway in macrophages to induce the polarization of macrophages to M2 type, thereby promoting tumor angiogenesis.

But our research still has certain limitations. The focus of our study was PTEN, TAMs, and tumor angiogenesis. However, the TME (Tumor microenvironment) is extremely complex in terms of its physiology and biochemical conditions and include a variety of cytokines related to angiogenesis. Moreover, TAMs can secrete many factors into the TME. Unfortunately, we did not choose the most influential one from the abundant cytokines to change the function of TECs, which is worthy of further study. At present, few studies have focused on the role of other factors or signaling pathways in the association between TAMs, PTEN, and ESCC angiogenesis. Therefore, it is necessary to further explore the complex relationship between these three factors to provide a comprehensive theoretical and experimental basis for the early diagnosis and clinical treatment of ESCC.

## Data Availability Statement

The original contributions presented in the study are included in the article/Supplementary Material. Further inquiries can be directed to the corresponding authors.

## Ethics Statement

The studies involving human participants were reviewed and approved by Ethics Committee of the First Affiliated Hospital of Zhengzhou University. The patients/participants provided their written informed consent to participate in this study.

## Author Contributions

KC and MS designed the research study and approved the final version of the manuscript for publication. CY conducted the research and wrote the paper. CC and QX collected and analyzed the data. XW, YS, XT, SW, HL, YL, and JS contributed to the research. All authors read and approved the final manuscript.

## Funding

This work was supported by grants from the National Natural Science Foundation of China (No. 81873455), National Natural Science Foundation of China Joint Fund Project (No. U1704173), and Henan Province Health Science and Technology Innovative Talents “51282” Project (Leading Talents) (2016).

## Conflict of Interest

The authors declare that the research was conducted in the absence of any commercial or financial relationships that could be construed as a potential conflict of interest.

## Publisher’s Note

All claims expressed in this article are solely those of the authors and do not necessarily represent those of their affiliated organizations, or those of the publisher, the editors and the reviewers. Any product that may be evaluated in this article, or claim that may be made by its manufacturer, is not guaranteed or endorsed by the publisher.

## Abbreviations

PTEN, phosphatase and tensin homolog; PI3K, phosphoinositide 3-kinase; TAM, tumor-associated macrophage; ESCC, esophageal squamous cell carcinoma; VEC, vein endothelial cell; TEC, tumor-associated vascular endothelial cell; MVD, microvessel density; FAM, Carboxyfluorescein; TME, Tumor microenvironment.
